# Utility and limitations of exome sequencing as a genetic diagnostic tool for conditions associated with pediatric sudden cardiac arrest/sudden cardiac death

**DOI:** 10.1186/s40246-015-0038-y

**Published:** 2015-07-19

**Authors:** Mindy H. Li, Jenica L. Abrudan, Matthew C. Dulik, Ariella Sasson, Joshua Brunton, Vijayakumar Jayaraman, Noreen Dugan, Danielle Haley, Ramakrishnan Rajagopalan, Sawona Biswas, Mahdi Sarmady, Elizabeth T. DeChene, Matthew A. Deardorff, Alisha Wilkens, Sarah E. Noon, Maria I. Scarano, Avni B. Santani, Peter S. White, Jeffrey Pennington, Laura K. Conlin, Nancy B. Spinner, Ian D. Krantz, Victoria L. Vetter

**Affiliations:** Department of Pediatrics, Perelman School of Medicine at the University of Pennsylvania, Philadelphia, PA USA; Division of Cardiology, The Children’s Hospital of Philadelphia, Philadelphia, PA USA; Division of Human Genetics, The Children’s Hospital of Philadelphia, Abramson Research Center, Room 1012G, 3615 Civic Center Blvd, Philadelphia, PA 19104 USA; Department of Biomedical and Health Informatics, The Children’s Hospital of Philadelphia, Philadelphia, PA USA; Department of Pathology & Laboratory Medicine, Perelman School of Medicine at the University of Pennsylvania, Philadelphia, PA USA; Division of Genomic Diagnostics, The Children’s Hospital of Philadelphia, Philadelphia, PA USA; Division of Oncology, The Children’s Hospital of Philadelphia, Philadelphia, PA USA; Present address: Department of Pediatrics, Cincinnati Children’s Hospital and Medical Center, and Department of Biomedical Informatics, College of Medicine, University of Cincinnati, Cincinnati, OH USA

## Abstract

**Background:**

Conditions associated with sudden cardiac arrest/death (SCA/D) in youth often have a genetic etiology. While SCA/D is uncommon, a pro-active family screening approach may identify these inherited structural and electrical abnormalities prior to symptomatic events and allow appropriate surveillance and treatment. This study investigated the diagnostic utility of exome sequencing (ES) by evaluating the capture and coverage of genes related to SCA/D.

**Methods:**

Samples from 102 individuals (13 with known molecular etiologies for SCA/D, 30 individuals without known molecular etiologies for SCA/D and 59 with other conditions) were analyzed following exome capture and sequencing at an average read depth of 100X. Reads were mapped to human genome GRCh37 using Novoalign, and post-processing and analysis was done using Picard and GATK. A total of 103 genes (2,190 exons) related to SCA/D were used as a primary filter. An additional 100 random variants within the targeted genes associated with SCA/D were also selected and evaluated for depth of sequencing and coverage. Although the primary objective was to evaluate the adequacy of depth of sequencing and coverage of targeted SCA/D genes and not for primary diagnosis, all patients who had SCA/D (known or unknown molecular etiologies) were evaluated with the project’s variant analysis pipeline to determine if the molecular etiologies could be successfully identified.

**Results:**

The majority of exons (97.6 %) were captured and fully covered on average at minimum of 20x sequencing depth. The proportion of unique genomic positions reported within poorly covered exons remained small (4 %). Exonic regions with less coverage reflect the need to enrich these areas to improve coverage. Despite limitations in coverage, we identified 100 % of cases with a prior known molecular etiology for SCA/D, and analysis of an additional 30 individuals with SCA/D but no known molecular etiology revealed a diagnostic answer in 5/30 (17 %). We also demonstrated 95 % of 100 randomly selected reported variants within our targeted genes would have been picked up on ES based on our coverage analysis.

**Conclusions:**

ES is a helpful clinical diagnostic tool for SCA/D given its potential to successfully identify a molecular diagnosis, but clinicians should be aware of limitations of available platforms from technical and diagnostic perspectives.

**Electronic supplementary material:**

The online version of this article (doi:10.1186/s40246-015-0038-y) contains supplementary material, which is available to authorized users.

## Background

The rapid development of genomic sequencing and techniques such as massively parallel next-generation sequencing has decreased cost, improved efficiency, and increased the clinical and research use of genetic testing [1, 2]. Exome sequencing (ES), or sequencing the protein-coding portions of a human genome, has become an increasingly utilized approach for investigating Mendelian disorders [3]. Studies report varying diagnostic ES success rates, ranging from 22.8 % [4] to 50 % [5]. As costs continue to decline, it is likely the use of whole genome-sequencing will increase [6]. The application of exome and genome-level sequencing raises many challenges both from a technical execution and diagnostic standpoint [7, 8], and the best use of this testing in clinical practice remains unclear [1]. The role of genetic testing as a tool to investigate cardiovascular disease has had increased focus in recent years [9].

Sudden cardiac arrest/death (SCA/D) is uncommon in the young and occurs in an estimated 2000 individuals under 25 years of age annually in the US [10]. Causes include inherited structural, functional, and electrical cardiac abnormalities [11–13]. There may be no significant previous medical history prior to the occurrence of SCA/D, and standard postmortem analysis may be unrevealing [12, 14, 15] in as many as 10-30 % of cases [16]. A pro-active family screening approach is important to provide life-saving treatment and to help identify other affected members due to the high association of genetic causes [12, 13]. More than 100 genes have been associated with SCA/D [11, 17]. Guidelines for genetic testing for channelopathies and cardiomyopathies, were published in 2011 [18].

Increased accessibility to ES warrants its examination as a possible front line diagnostic tool for inherited conditions associated with SCA/D. Compared with targeted gene sequencing and comprehensive panels specific for disease, which are currently available [11], there are important differences to consider with ES. The workflow and challenges of completing ES are described in detail by Bamshad et al. [3]. Importantly, only 1-2 % of the human genome contains protein–coding sequences [3, 6, 19]; thus, these regions must undergo an exon-targeting “*capture*” process before being sequenced. Traditional sequencing methods for individual genes or panels do not require this step.

A second consideration is determining the “*coverage*” of these captured regions. Coverage (also known as “*depth*” of sequencing) refers to how many times a nucleotide meeting criteria for being a high-quality base is represented in a random collection of raw sequences [20]. This helps differentiate sequencing errors from a true sequence variant; the higher the coverage, the more likely the captured base is accurate and not a false read due to technical errors. For example, a captured base “T” with 20x coverage means that base is represented at least 20 times at that position in multiple raw sequences. Of note, “coverage”, in addition to meaning *depth* of sequencing, may also refer to the general *proportion* of bases covered in genomic sequence at a specific depth [21]. For example, an exon with 90 % coverage at 20x means 90 % of the bases in that exon are represented at least 20 times on multiple raw sequences. In our manuscript, *depth* of sequencing will refer to the number of times a nucleotide is represented, and *coverage* will refer to the general proportion of genomic sequence covered unless otherwise specified.

**Fig. 1:**
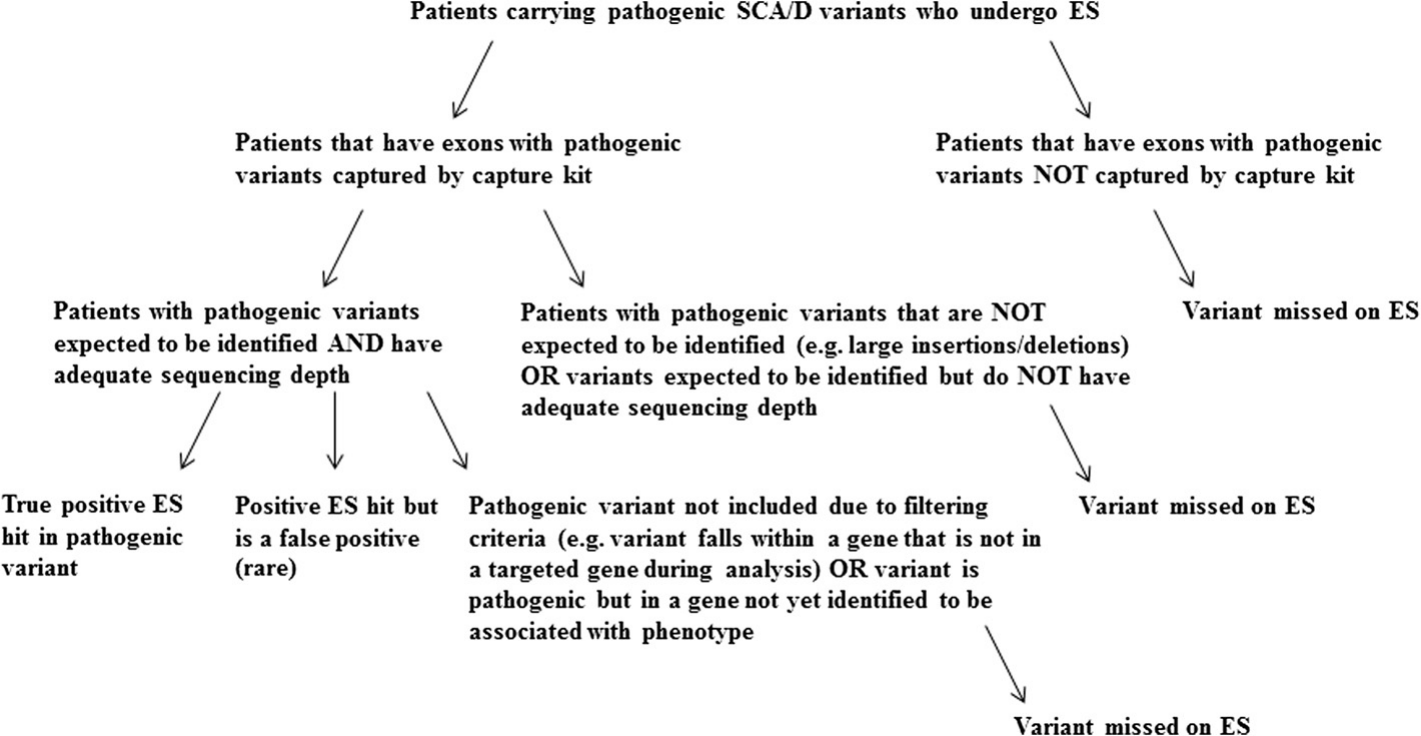
General schematic of hypothetical analysis of patients carrying pathogenic SCA/D variants who undergo ES

Figure 1 outlines a basic schematic of a hypothetical analysis of patients carrying pathogenic SCA/D variants via ES and the steps that potentially can lead to missed calls. Though there is much debate regarding the use and implications of ES in a clinical setting, there is little information available regarding the capture and coverage of cardiac genes commonly found within comprehensive panels, which has implications for diagnostics and ability to find potential pathogenic mutations. The main objective of this study was to investigate the utility of ES as a potential diagnostic tool by investigating capture and depth of coverage of a group of targeted genes related to SCA/D.

## Methods

### Study population

This research study was approved by The Children’s Hospital of Philadelphia (CHOP) Institutional Review Board (IRB). Samples from 102 pediatric individuals were enrolled under an IRB approved protocol of informed consent at The Children’s Hospital of Philadelphia and de-identified. Detailed demographic information of individuals was not readily available due to the de-identification process. Of these 102 patients, thirteen had known molecular diagnosis for SCA/D, 59 had known molecular etiologies for other conditions including hearing loss, intellectual disability and mitochondrial disease (all known diagnoses were identified by Clinical Laboratory Improvement Amendments certified laboratories), and 30 individuals did not have known molecular etiologies for SCA/D.

### Exome capture, sequencing, and bioinformatics

Peripheral blood from patients was collected in sterile EDTA tubes (BD vacutainer) at Phlebotomy, Children’s Hospital of Philadelphia. Blood tubes were stored immediately at 4 °C and Genomic DNA (gDNA) was manually extracted using standard procedures with the Gentra Puregene Blood Kit Plus (Qiagen, 158489). gDNA quality was assessed on an agarose gel, Nanodrop spectrophotometer and quantified via the Qubit system. 3-6ug of gDNA from each sample were prepared and sent to the Beijing Genomics Institute (BGI) facility at CHOP. Exome capture was done with Agilent SureSelect V4, and whole-exome sequencing was completed on Illumina Hi-Seq 2000 sequencers at an average coverage depth of 100X. Sequencing reads were obtained in FASTQ format and were examined via the Pediatric Genetic Sequencing Project (PediSeq) exome sequence coverage analysis pipeline.

The sequence reads were mapped to human genome assembly, GRCh37.p10, using Novoalign (V3.00.02) (www.novocraft.com), which has been shown to optimize alignment [22]. Coverage statistics per exons in the SCA/D genes bed file were generated using GATK Depth of Coverage tool version 2.2. Quality control steps to filter out poorly mapped reads included minBaseQuality 20/minMapping Quality 20 settings during variant calling and removal of variants with a minimum depth of coverage of less than 20 reads. Multi-sample variant calling was not done as patients are clinically evaluated and analyzed independently. Refer to Additional file 1 for further details regarding the sequencing protocol and data processing.

The primary aim of this study was to evaluate the adequacy of depth of sequencing, coverage of targeted SCA/D genes, and platform efficacy, not to identify individual molecular diagnoses. Therefore, the main *coverage* analysis was completed on the first 72 samples, regardless of their underlying clinical findings and molecular etiologies. Of these 72 samples, additional analysis (described at the end of the methods section) was completed on the 13 cases with known molecular causes for SCA/D. In addition, *diagnostic* analysis was performed on 30 patients without known causes for their SCA/D. 15 of these 30 patients (50 %) had variable prior genetic workup that was non-diagnostic.

A list of 103 genes associated with SCA/D (Table 1) was manually curated and used as a primary filter for analysis. To determine the full scope of variants that could be missed using ES, the first step was to determine the total number of exons present within the targeted genes, and within those exons, determine how many variants have been previously reported and suspected to be pathogenic. Information regarding reported variants can be found in the Human Gene Mutation Database (HGMD), a comprehensive database that aims to compile nuclear gene germline mutations that have been associated with human disease [23]. We recognize not all variants reported in HGMD may be considered pathogenic, but for the purposes of this study any variant reported in HGMD was considered a potentially disease causing change that would require further review if picked up on ES.

**Table 1:** Curated List of 103 Genes Associated with SCA/D

*ABCC9*	*CAV3*	*ELN*	*KCNE3*	*MYH6*	*RBM20*	*TCAP*
*ACTA2*	*CBS*	*EMD*	*KCNH2*	*MYH7*	*RYR2*	*TGFB3*
*ACTC1*	*COL3A1*	*EYA4*	*KCNJ2*	*MYL2*	*SCN1B*	*TGFBR1*
*ACTN2*	*COL5A1*	*FBN1*	*KCNJ5*	*MYL3*	*SCN3B*	*TGFBR2*
*AKAP9*	*COL5A2*	*FBN2*	*KCNJ8*	*MYLK2*	*SCN4B*	*TMEM43*
*ANK2*	*CRYAB*	*FHL2*	*KCNQ1*	*MYOZ2*	*SCN5A*	*TMPO*
*ANKRD1*	*CSRP3*	*FKTN*	*KRAS*	*NEXN*	*SDHA*	*TNNC1*
*BAG3*	*CTF1*	*GATAD1*	*LAMA4*	*NRAS*	*SGCD*	*TNNI3*
*BRAF*	*DES*	*GLA*	*LAMP2*	*PKP2*	*SHOC2*	*TNNT2*
*CACNA1B*	*DMD*	*GPD1L*	*RPSA*	*PLN*	*SLC25A4*	*TPM1*
*CACNA1C*	*DPP6*	*HRAS*	*LDB3*	*PRKAG2*	*SLC2A10*	*TTN*
*CACNA2D1*	*DSC2*	*JPH2*	*LMNA*	*PSEN1*	*SMAD3*	*TTR*
*CACNB2*	*DSG2*	*JUP*	*MAP2K1*	*PSEN2*	*SNTA1*	*VCL*
*CALR3*	*DSP*	*KCNE1*	*MYBPC3*	*PTPN11*	*SOS1*	
*CASQ2*	*DTNA*	*KCNE2*	*MYH11*	*RAF1*	*TAZ*	

We then evaluated the proportion of different variant types present within the 2,190 exons. This was critical as changes such as large insertions or deletions do not have specific genomic coordinates, and locating these changes can be problematic due to current limitations of ES technology. As large insertions and deletions were unlikely to be picked up with ES without separate and additional computational analyses, they were not included in the final analysis.

The next round of analysis focused on three main aspects: 1) Examining how well the exons in the targeted genes were *captured* on the Agilent SureSelect V4 platform, 2) Of the exons that were captured, how adequate was the *depth* of sequencing of these exons, using 20x (when a nucleotide on average is represented at least 20 times in a group of random raw sequences) as our standard for defining adequate depth of sequencing, and 3) Of the captured exons, what proportion of the exons met criteria for adequate sequencing *coverage* (percentage of bases within the exons that are sequenced at an appropriate read depth, which in this case was 20x). Coverage scores of all 2,190 exons were obtained for each sample individually, and then data for each exon was averaged across all samples.

Although the primary objective of this study was to evaluate the adequacy of depth of sequencing and coverage of targeted SCA/D genes and not for primary diagnosis, all patients enrolled in the study who had known molecular etiologies for SCA/D were evaluated with the project’s variant analysis pipeline to determine if the molecular etiologies could be successfully identified. Project members completing this analysis were blinded to the known molecular diagnosis of the patients to avoid bias during the evaluation process. As this group of patients was relatively small (*n* = 13), additional variants within the targeted genes associated with SCA/D were selected and evaluated for depth of sequencing and coverage to determine how well a random number of potentially disease related mutations would be picked up on ES. One hundred variants reported in the Human Gene Mutation Database (HGMD) that were within the 103 genes associated with SCA/D were randomly selected for analysis to ensure a varied distribution. Statistics regarding the capture and depth of sequencing on those specific variants were generated using GATK Depth of Coverage tool version 2.2.

Beyond the 72 individuals used in the primary coverage analysis, the additional 30 patients with a history of SCA/D but no known molecular causes were analyzed using the same variant analysis pipeline to determine a diagnostic yield. Results were deemed “positive” if there were variants in genes related to SCA/D categorized as likely pathogenic or pathogenic, “uncertain” if there were only variants in genes related to SCA/D categorized as variants of uncertain significance (VUS), and “negative” if there were no VUS, suspected pathogenic, or pathogenic variants identified in genes related to SCA/D.

## Results

**Fig. 2:**
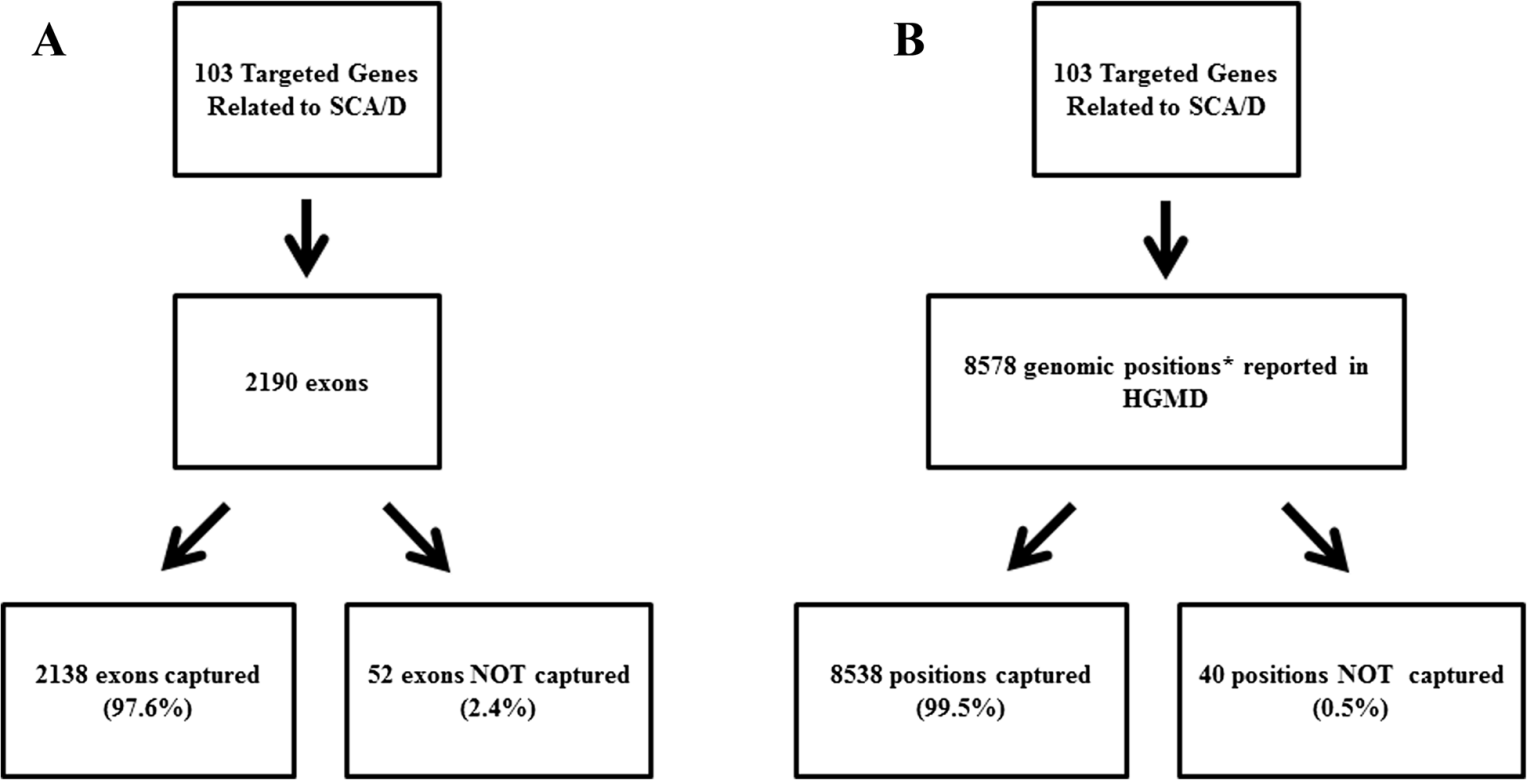
**a**, Distribution of captured versus non-captured exons within 103 targeted genes associated with SCA/D. **b**, Distribution of unique genomic positions with reported pathogenic variants in Human Gene Mutation Database (HGMD) that fall within the captured and non-captured exons in the targeted 103 genes associated with SCA/D. Genomic position (*) refers to the location of variant on a chromosome

**Fig. 3:**
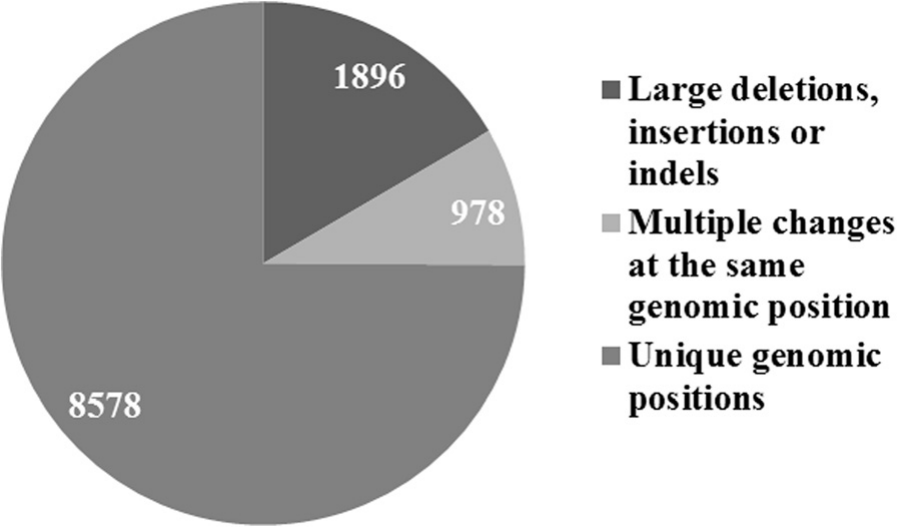
Distribution of reported Human Gene Mutation Database (HGMD) variants within the exons of the 103 genes associated with SCA/D list (*n* = 11,452)

Within the 103 targeted genes associated with SCA/D, there were 2,190 exons present (Fig. 2a). Within these exons, the total number of reported variants suspected to be disease causing in HGMD was 11,452 (Fig. 3). Of the 11,452 variants reported in HGMD in our targeted exons, 1,896 (16.6 %) were large deletions or insertions, and 9,556 (83.4 %) were variants with a reported genomic position (Fig. 3). Of these variants with a coordinate, 8,578 (89.8 %) were associated with a unique genomic position (e.g., base “T” at position “X”), and 978 (10.2 %) represented the number of positions with multiple base pair changes reported at the same position (e.g., both base “T” and base “A” changes reported at position “X”) (Fig. 3). Using our specific capture kit and sequencing technology, 2,138 out of 2,190 exons were captured (97.6 %), and 52 out of the 2,190 exons (2.4 %) were not captured (Fig. 2a) during the sequencing process. Within the captured exons, there were 8538 genomic positions (99.5 % of 8578 total genomic positions) published HGMD variants (Fig. 2b).

We were also interested in the proportion of unique genomic positions falling within exons that were captured but had poor *depth* of sequencing and thus potentially poor *coverage*. We considered an exon to have inadequate coverage (“not covered” or “no coverage”) when less than 40 % of the bases within the exon met criteria for having sequence depth of at least 20x. Within the 72 samples, the number of unique genomic positions falling within captured exons that fell in this category ranged from 44 to 587, with a median of 374. Averaged across all 72 samples, there were approximately 344/8538 (4 %) unique genomic positions falling within captured exons that had less than 40 % of bases sequenced at 20x depth (“no coverage”).

**Fig. 4:**
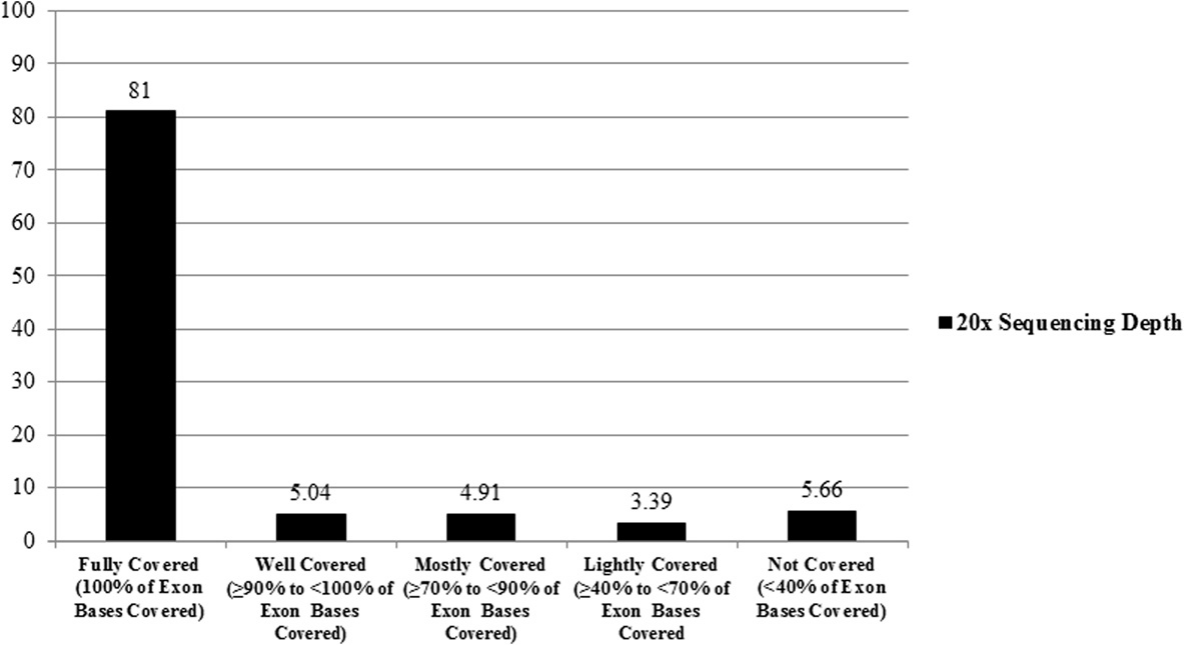
Average Percentage of Exon Coverage Categories of Targeted SCA/SCD Genes at 20x sequencing depth

In contrast, Fig. 4 delineates the proportion of exons that met criteria for “adequate” sequencing coverage in the captured exons. The exon proportions were separated into different categories based on what percentage of bases within the exons had adequate depth of sequencing at 20x. We considered an exon “fully covered” if 100 % of the bases in that exon were covered at 20x, “well covered” if ≥90 % to less than 100 % of bases in the exon were covered at 20x, “mostly covered” if ≥70 % to less than 90 % of the bases in the exon were covered at 20x, lightly covered if ≥40 % to less than 70 % of the bases in the exon were covered at 20x, and “not covered” if <40 % of the bases in the exon were covered at 20x. Proportions for each of the coverage categories were obtained for samples individually, and then they were averaged across all samples. On average, 81 % of the total exons were fully covered, 5.04 % of exons were well covered, 4.91 % of exons were mostly covered, 3.39 % of exons were lightly covered, and 5.66 % of exons were not covered.

**Fig. 5:**
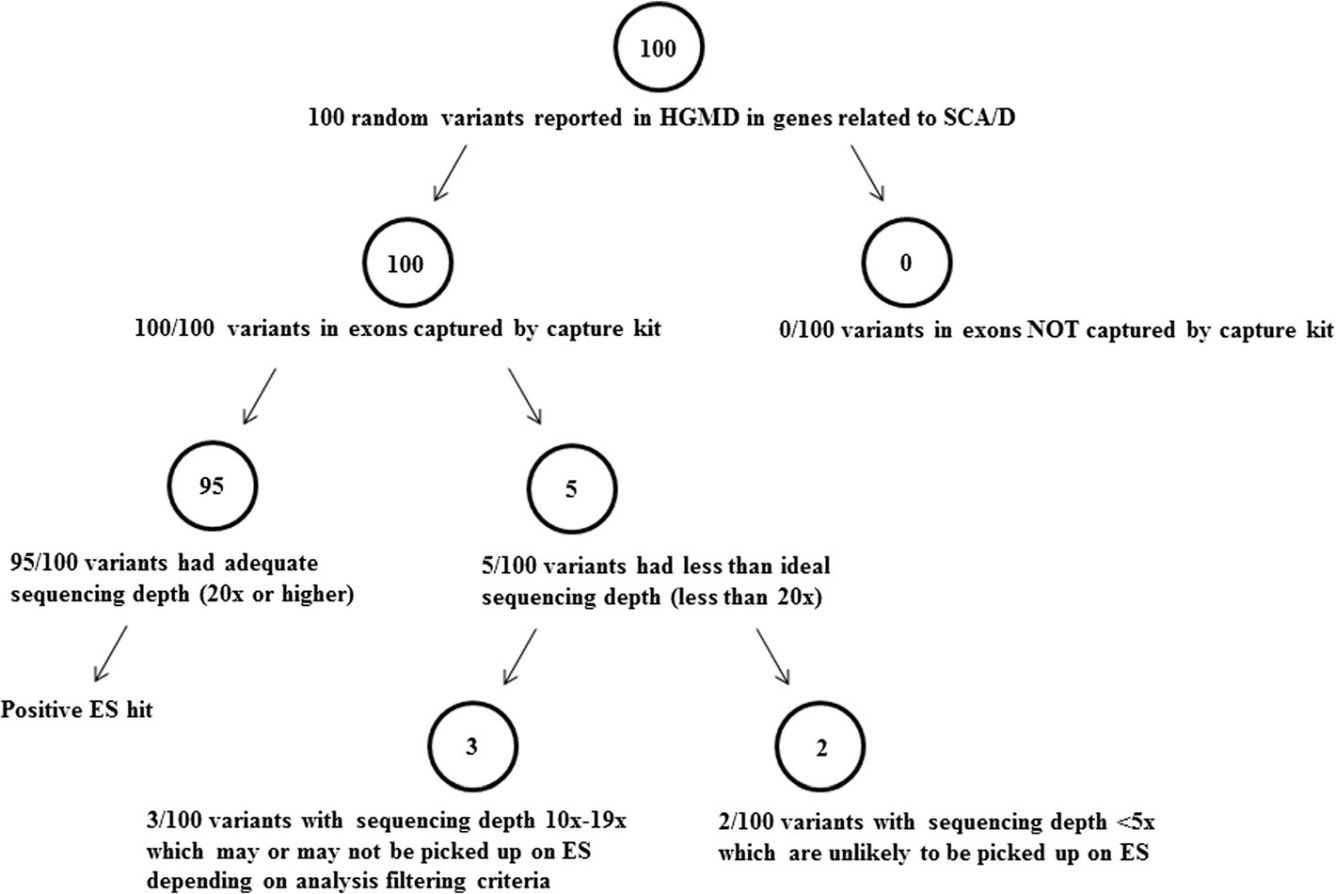
Schematic analysis of 100 random pathogenic variants, suspected pathogenic variants, or variants of uncertain significance (VUS) reported in Human Gene Mutation Database (HGMD) in genes related to SCA/D

All patients enrolled in the study who had known molecular etiologies for SCA/D (*n* = 13) were also evaluated to see if the causal molecular etiologies could be identified using the project’s variant analysis ES pipeline. Of these patients, 13/13 pathogenic variants previously called by CLIA certified laboratories were accurately identified. Due to a small sample size, additional coverage analysis was performed on 100 random variants within the 103 SCA/D genes reported in HGMD to evaluate how well these would be picked up on ES (see Additional file 2 for variant list). All 100 variants were captured by the capture kit (Fig. 5). The next step was to address adequate depth of sequencing. Using 20x as the standard for defining adequate depth of sequencing, 95/100 variants met this criteria, and the remaining 5/100 variants had a sequencing depth of less than 20x (Fig. 5; Table 2). Of these variants, 3 had sequencing depths of 10x-19x, and 2 had a sequencing depth of less than 5x (Fig. 5; Table 2).

**Table 2:** Randomly selected variants potentially associated with disease within targeted SCA/D genes with less than 20x sequencing depth

Gene	OMIM^a^ #	Phenotype	Variant	Sequencing depth
*COL3A1*	120180	Ehlers-Danlos syndrome IV	c.3230G > T	12
*CTF1*	600435	Cardiomyopathy, dilated	c.274G > A	2
*PKP2*	602861	Arrhythmogenic right ventricular dysplasia	c.1237C > T	11
*SDHA*	600857	Complex II deficiency & Dilated cardiomyopathy, 1GG	c.1664G > A	1
*TMPO*	188380	Cardiomyopathy, dilated	c.2068C > T	13

^a^Online Mendelian Inheritance in Man

An additional 30 patients with history of SCA/D and no known molecular etiology were also analyzed using the same variant analysis pipeline. Results revealed a positive causative finding in 5/30 cases (17 %, Table 3), uncertain results in 16/30 cases (53 %), and negative results in 9/30 (30 %).

**Table 3:** Pathogenic or likely pathogenic variants identified on ES in samples without prior known molecular diagnosis

Gene	Phenotype of gene	Variant	Protein change	Zygosity
*SCN5A*	BrS^a^, DCM^b^, Familial atrial fib, Long QT	c.4867C > T	p.Arg1623*	Heterozygous
*DSP*	ARVD^c^, DCM	c.928dupG	p.Glu310Glyfs*13	Heterozygous
*KCNE1*	Long QT	c.226G > A	p.Asp76Asn	Heterozygous
*KCNH2*	Long QT, Short QT	c.1750G > A	p.Gly584Ser	Heterozygous
*KCNQ1*	Familial atrial fib, Long QT, Short QT	c.513C > A	p.Tyr171*	Heterozygous

^a^Brugada syndrome, ^b^Dilated cardiomyopathy, ^c^Arrhythmogenic right ventricular dysplasia

## Discussion

Our results revealed a number of findings demonstrating the strengths and limitations of using ES as a diagnostic tool. First, there was a fair percentage (16.6 %) of variants within the targeted genes reported in HGMD (large deletions or insertions without genomic positions) that would not be expected to be seen with ES due to the limitations of current technology. Though the ability to identify such changes will likely improve with better technology, clinicians should be aware of the types of platforms being used to capture exonic sequence as well as the limitations of sequence and variant calling technologies to successfully sequence and identify certain types of mutations. In our analysis, amongst the variants reported that have a genomic position, the majority (89.8 %) were unique genomic positions. We were primarily interested in the number of unique genomic positions since presumably a position that has good depth of sequencing would be covered regardless of the base pair change at that location.

Second, the results demonstrate that the majority of exons (97.6 %) within the targeted SCA/SCD genes were captured with our specific capture kit. The remaining small portion of exons (2.4 %) were not captured primarily because our capture kit did not target these exons, so these areas would not be expected to be picked up even on subsequent sample runs. There are regions of DNA that can be difficult to capture due to the inherent sequence/structure (e.g., repetitive and GC rich regions) resulting in a technical inability to target and capture every exon in the human genome. Thus, the proportion of unique genomic positions reported in HGMD falling within these non-captured regions would be potentially missed. However, within the non-captured exons, there were only 40 genomic positions (0.5 % of 8578 total genomic positions) with published HGMD variants that would not be expected to be picked up (Fig. 2b). This reflects the process was able to capture the majority of exons with minimal reported HGMD positions missed due to capture issues alone. It is important to be mindful that the goal of individual capture kits is to obtain consistent coverage on the desired targets, but capture and coverage of the non-targeted regions will vary depending on the run due to the limitations of technology.

Aside from the initial capture process, variants could also be missed due to inadequate depth of sequencing. For example, 2 out of 2,190 exons were targeted for capture across all 72 samples but had zero depth of sequencing (nucleotides in those exons were represented zero times on sequences). Within captured exons of individual samples, we were most interested in the proportion of reported HGMD unique genomic positions falling within exons meeting criteria for “no coverage” because these have the potential to be missed on ES analysis. There was a wide range within individual samples, but overall the average proportion of positions in non-covered regions was relatively small (4 %). When examining the proportion of the total number of exons that fell under different coverage categories at 20x sequencing depth, there was a wide distribution with the majority (81 %) of exons being fully covered and a minority (5.66 %) of exons not covered at all. The middle categories of well covered, mostly covered, and lightly covered (13.34 % of exon proportions in aggregate) still made up a fair portion of exons without complete coverage in our targeted gene list. Our results highlight the variability that exists with coverage using ES, and poorly covered regions, as well as moderately covered regions would likely benefit from improving coverage enrichment of kits in these areas. In spite of these limitations in coverage, it is notable that all of the known pathogenic mutations in the 13 samples with confirmed molecular etiologies for SCA/D were identified by using ES (Table 4), and causal findings were identified in 17 % of an additional set of 30 SCA/D patients without known molecular etiologies referred for primary analysis (Table 3). While the main purpose of this study was not to identify diagnostic yields of a particular cohort, these results are reflective of how a primary diagnosis can successfully be made using ES.

**Table 4:** Pathogenic variants identified on ES in samples with known molecular diagnosis

#	Gene	Phenotype of gene	Variant	Protein change	Zygosity	Change found
1	*KCNH2*	Long QT, Short QT	c.1882G > A	p.Gly628Ser	Heterozygous	Yes
2	*KCNQ1*	Familial atrial fib, Long QT, Short QT	c.1552 C > T	p.Arg518^a^	Heterozygous	Yes
3	*KCNH2*	Long QT, Short QT	c.1838C > T	p.Thr613Met	Heterozygous	Yes
4	*SCN5A*	BrS^a^, DCM^b^, Familial atrial fib, Long QT	c.4978A > G	p.Ile1660Val	Heterozygous	Yes
5	*KCNQ1*	Familial atrial fib, Long QT, Short QT	c.704 T > A	p.Ile235Asn	Heterozygous	Yes
6	*MYL2*	HCM^c^	c.173G > A	p.Arg58Gln	Heterozygous	Yes
7	*KCNE1*	Long QT	c.226G > A	p.Asp76Asn	Heterozygous	Yes
8	*KCNQ1*	Familial atrial fib, Long QT, Short QT	c.1140G > T	p.Arg380Ser	Heterozygous	Yes
9	*MYH7*	DCM, HCM, LVNC^d^	c.2572C > T	p.Arg858Cys	Heterozygous	Yes
10	*MYL2*	HCM	c.173G > A	p.Arg58Gln	Heterozygous	Yes
11	*TTN*	DCM, HCM	c.59216 T > A; c.94578delT	p.Val19664Glu; p.Thr31451Thrfs^a^9	Compound Heterozygous	Yes
12	*FBN1*	Marfan syndrome	c.2347 A > C	Asn783His	Heterozygous	Yes
13	*ACTC; TTN*	DCM, HCM, LVNC; DCM, HCM	ACTC: c.806 T > C; TTN: c.11323 G > A	Ile269Thr; Ala3775Thr	Heterozygous	Yes

^a^Brugada syndrome, ^b^Dilated cardiomyopathy, ^c^Hypertrophic cardiomyopathy, ^d^Left ventricular noncompaction

We took an additional step to evaluate 100 random variants that have been potentially associated with disease in our targeted SCA/D genes across all individual samples (Additional file 2). The capture of these variants was ideal with all 100 variants successfully captured by the capture kit (Fig. 5). In terms of coverage, the majority (95/100) of the variants were sequenced adequately at 20x and thus would have been picked up on ES. Of the remaining variants with sequencing depths less than 20x, only 2 had sequencing depths less than 5x and would be likely to be missed on ES (Fig. 5; Table 2). Although 20x is often the ideal standard for sequencing depth, many analysis pipelines include variants with lower cutoffs; thus, depending on what analysis protocols are used, up to 5 % of these particular variants may not have been picked up on ES due to sequencing depth. In sum, although at this time ES does not fully cover every base pair at 20x within our targeted genes, the likelihood of a missed variant due to coverage issues remains small.

### Limitations

There are a number of limitations to this study that should be considered. First, coverage data from this analysis was limited to the focused gene list that was curated by our team. It is recognized that all genes associated with SDA/D are not included in this list and that new genes are frequently discovered as updated information becomes available. Since the completion of this study, we have added additional genes associated with SCA/D to our list. A pathogenic variant may exist in a patient, but it will not be picked up on ES if the gene has not yet been associated with that particular phenotype and/or human disease. Genes are uniquely different in terms of genomic location, size, number of exons, repeat regions and GC rich regions, and other characteristics that can potentially affect the ability to capture exons and have appropriate coverage. As new genes related to SCA/SCD are discovered, it is important to consider how these might be best captured and what is necessary to improve coverage. Sims et al. reviewed in depth issues of sequencing coverage [21]. Definitions for what constitutes specific coverage “levels” also vary among institutions and should be taken into account when performing ES analysis. Additionally, available exome capture kits differ between vendors, though functionality has been found to be generally equal [3], and reproducibility varies with each use, even when using the same kits. Factors such as level of enrichment, genomic library detail, and consistency of captured targets play a role [3].

Finally, as the goal of ES is to identify those variants that may be potentially pathogenic and disease causing, it is equally important to have efficient strategies and appropriate variant analysis pipelines. Without a solid analysis pipeline, consistent capture and high coverage alone is not adequate to detect meaningful variants. A pathogenic variant may be present, but if it is not targeted in analysis it may not be found depending on the filtering parameters. This consideration will continue to be of importance even as the use of genome level sequencing potentially increases beyond ES in the future. Genome sequencing would allow changes beyond the coding regions to be identified, and it would not face the technical challenges seen in ES such as exon capture and coverage. However, the number of potential variants to analyze would increase tremendously and would be require more sophisticated analysis pipelines to filter and identify disease-causing changes.

## Conclusions

Given the high genetic heterogeneity of conditions leading to SCA/SCD, genomic sequencing has the potential to provide invaluable clinical information to high-risk families and clinicians and to help identify at-risk individuals in whom management can help to prevent future SCA/SCD. Our results revealed both the abilities and limitations of using ES as a tool to evaluate genes related to conditions associated with SCA/SCD. Although ES is not fully comprehensive for our targeted genes at this time compared to traditional single or multi-gene panels, the majority of exons were still captured with commercially available kits and were also fully covered on average at 20x sequencing depth. Also, the proportion of HGMD unique genomic positions reported within poorly covered exons remained small. Exonic regions with less coverage reflect the need to enrich these regions to improve coverage. Despite limitations in coverage, our results show ES has a strong potential to pick up molecular changes as we were able to identify 100% of cases with known molecular etiologies for SCA/D in our small cohort. Additionally, in a cohort of 30 patients without a known molecular etiology for their SCA/D we were able to identify a likely etiology in 17 %. We were also able to demonstrate at least 95 % of a number of randomly selected HGMD reported variants would have been picked up on ES as well based on coverage analysis. Overall, ES is a helpful genetic diagnostic tool for SCA/SCD in the clinical setting given its potential to successfully reveal a molecular diagnosis, but clinicians should be aware of limitations of currently available platforms from both a technical and diagnostic perspective.

## Additional files

Additional file 1:
**Expanded methods.**
Additional file 2:
**100 random variants potentially associated with disease in our targeted SCA/D genes selected for capture and coverage analysis across all individual samples.**

## References

[1] Jamal SM, Yu J, Chong JX, Dent KM, Conta JH, Tabor HK, Bamshad MJ. Practices and Policies of Clinical Exome Sequencing Providers: Analysis and Implications. Am J Med Genet A. 2013;161A:n/a-n/a. 10.1002/ajmg.a.35942 PMC3708985 23610049

[2] Interpreting Secondary Cardiac Disease Variants in an Exome Cohort. Circ Cardiovasc Genet. 2013;6:337–46. 10.1161/CIRCGENETICS.113.000039 PMC3887521 23861362

[3] Bamshad MJ, Ng SB, Bigham AW, Tabor HK, Emond MJ, Nickerson DA (2011). Exome sequencing as a tool for Mendelian disease gene discovery. Nat. Rev. Genet..

[4] Atwal PS, Brennan M, Cox R, Niaki M, Platt J, Homeyer M (2014). Clinical whole-exome sequencing: are we there yet?. Genet. Med..

[5] Clinical application of exome sequencing in undiagnosed genetic conditions. J Med Genet. 2012;49:353–61. 10.1136/jmedgenet-2012-100819 PMC3375064 22581936

[6] Wang Z, Liu X, Yang B, Gelernter J (2013). The Role and Challenges of Exome Sequencing in Studies of Human Diseases. Front Genet.

[7] Yang Y, Muzny DM, Reid JG, Bainbridge MN, Willis A, Ward PA (2013). Clinical Whole-Exome Sequencing for the Diagnosis of Mendelian Disorders. N Engl J Med.

[8] Ng SB, Turner EH, Robertson PD, Flygare SD, Bigham AW, Lee C (2009). Targeted capture and massively parallel sequencing of 12 human exomes. Nature.

[9] Arndt A, MacRae CA (2014). Genetic testing in cardiovascular diseases. Curr. Opin. Cardiol..

[10] Pediatric Sudden Cardiac Arrest. Pediatrics. 2012;129:e1094-e1102. 10.1542/peds.2012-0144 22451713

[11] Wilde AAM, Behr ER (2013). Genetic testing for inherited cardiac disease. Nat Rev Cardiol.

[12] Contribution Of Inherited Heart Disease To Sudden Cardiac Death In Childhood. Pediatrics. 2007;120:E967-E973. 10.1542/peds.2006-3751 17908752

[13] Yield of Molecular and Clinical Testing for Arrhythmia Syndromes: Report of 15 Years' Experience. Circulation. 2013;128:1513–21. 10.1161/CIRCULATIONAHA.112.000091 23963746

[14] Kauferstein S, Kiehne N, Jenewein T, Biel S, Kopp M, König R (2013). Genetic analysis of sudden unexplained death: A multidisciplinary approach. Forensic Sci. Int..

[15] Kumar S, Peters S, Thompson T, Morgan N, Maccicoca I, Trainer A (2013). Familial cardiological and targeted genetic evaluation: Low yield in sudden unexplained death and high yield in unexplained cardiac arrest syndromes. Heart Rhythm.

[16] Tester DJ, Ackerman MJ (2007). Postmortem Long QT Syndrome Genetic Testing for Sudden Unexplained Death in the Young. J. Am. Coll. Cardiol..

[17] OMIM. Online Mendelian Inheritance in Man, OMIM®. Johns Hopkins University, Baltimore, MD. World Wide Web URL: http://www.omim.org.

[18] HRS/EHRA Expert Consensus Statement on the State of Genetic Testing for the Channelopathies and Cardiomyopathies: This document was developed as a partnership between the Heart Rhythm Society (HRS) and the European Heart Rhythm Association (EHRA). Europace. 2011;13:1077–109. 10.1093/europace/eur245 21810866

[19] Krawitz P, Mundlos S (2011). Strategies for exome and genome sequence data analysis in disease-gene discovery projects. Clin. Genet..

[20] Raymond C, Raymond C, Aravind L (2001). Initial sequencing and analysis of the human genome. Nature.

[21] Sims D, Sudbery I, Ilott NE, Heger A, Ponting CP (2014). Sequencing depth and coverage: key considerations in genomic analyses. Nat. Rev. Genet..

[22] Li H. Aligning Sequence Reads, Clone Sequences And Assembly Contigs With BWA-MEM. 03 2013. 1303.

[23] Stenson PD, Mort M, Ball EV, Shaw K, Phillips AD, Cooper DN (2013). The Human Gene Mutation Database: building a comprehensive mutation repository for clinical and molecular genetics, diagnostic testing and personalized genomic medicine. Hum. Genet..

