# Supplementary material for: Utility and limitations of exome sequencing as a genetic diagnostic tool for conditions associated with pediatric sudden cardiac arrest/sudden cardiac death
Source: Hum Genomics. 2015 Jul 19;9(1):15. doi: 10.1186/s40246-015-0038-y (PMC4506570; doi:10.1186/s40246-015-0038-y)
Supplement: Additional file 1: — Expanded methods. [file 40246_2015_38_MOESM1_ESM.docx]

ADDITIONAL FILE 1

EXPANDED METHODS

**Sequencing Protocol**

Whole exome library preparation is based on modification of the protocol using the Agilent SureSelect Whole Exome, version 4 kit (51 MB target size). Three micrograms of genomic DNA are sheared with a Covaris sonicator to an average size of 150-200 bp (yield and size distribution of this and other library steps are performed by analysis on a Caliper LabChip GX System). Subsequent steps of the pre-capture workflow include end repair, addition of an “A” overhang at the 3’ end, ligation of the indexing-specific adaptor, and amplification of the library. Following each step, library material is purified using Agencourt Ampure XP beads. Concentration of the amplified library is then assessed and adjusted to an amount appropriate for the capture hybridization. The amplified library is mixed with hybridization buffer components, blocking oligos, RNase inhibitor and the library of whole exome “baits” and incubated for 24 hours or more at 65 ^o^C. After hybridization, the mixture is bound to Dynabeads MyOne Streptavidin T1 and subjected to two rounds of washing to retain only specific hybridization products. The resulting capture library is then amplified, using a minimum amount of cycles to reduce the number of duplicate reads. During this amplification, bar codes are added to individual libraries, which are purified with Ampure XP beads. Size and yield of the bar-coded libraries are assessed on the LabChip GX, with an expected distribution of 300-400 bp. Concentration of each library is measured either from the LabChip GX output or via real-time PCR. Pools of indexed samples are then created in preparation for cluster generation and 100 bp x 100 bp paired end sequencing on the Illumina HiSeq 2500s. The degree of multiplexing per lane is determined by the desired coverage. For whole exome 100X coverage, four samples can be multiplexed per lane.

**Sequence Data Processing**

Sequence read alignments are completed using Novoalign (V3.00.02) against the human reference genome GRCh37.p10 (http://www.novocraft.com). If the percentage of reads aligned is greater than 85%, it is considered sufficient to proceed to variant calling. Both SNP and InDel calls are then made using the Broad Institute’s GATK (McKenna et al. 2010) best practices for variant detection. SNPEff (Cingolani et al. 2012) is used to annotate the variants using publically available information from RefSeq (hg19) and Ensembl (GRCh37.66). The overall data management and processing, from fastq to vcf, is governed by a Pegasus workflow (Pegasus workflow management system, Deelman et al, 2005).
