# Supplementary material for: Utility and limitations of exome sequencing as a genetic diagnostic tool for conditions associated with pediatric sudden cardiac arrest/sudden cardiac death
Source: Hum Genomics. 2015 Jul 19;9(1):15. doi: 10.1186/s40246-015-0038-y (PMC4506570; doi:10.1186/s40246-015-0038-y)
Supplement: Additional file 2: — 100 random variants potentially associated with disease in our targeted SCA/D genes selected for capture and coverage analysis across all individual samples. [file 40246_2015_38_MOESM2_ESM.docx]

ADDITIONAL FILE 2

SUPPLEMENTARY TABLE: 100 random variants potentially associated with disease in our targeted SCA/D genes selected for capture and coverage analysis across all individual samples.

| **GENE** | **OMIM* #** | **HGVS**^†^ **Nucleotide** | **HGVS Protein** | **Phenotype**^‡^ |
| --- | --- | --- | --- | --- |
| *ABCC9* | 601439 | c.4640C>T | p.T1547I | Atrial fibrillation |
| *ACTA2* | 102620 | c.350A>C | p.N117T | Thoracic aortic aneurysms and dissections |
| *ACTC1* | 102540 | c.301G>A | p.E101K | Cardiomyopathy, hypertrophic |
| *ACTN2* | 102573 | c.1484C>T | p.T495M | Cardiomyopathy, hypertrophic |
| *AKAP9* | 604001 | c.4709C>T | p.S1570L | Long QT syndrome |
| *ANK2* | 106410 | c.11716C>T | p.R3906W | Cardiac arrhythmia |
| *ANKRD1* | 609599 | c.313C>T | p.P105S | Cardiomyopathy, dilated |
| *BAG3* | 603883 | c.652C>T | p.R218W | Cardiomyopathy, dilated |
| *BRAF* | 164757 | c.107C>G | p.S36C | Cardiomyopathy, hypertrophic |
| *CACNA1C* | 114205 | c.1468G>A | p.G490R | Brugada syndrome (shorter-than-normal QT interval) |
| *CACNA2D1* | 114204 | c.2867C>A | p.S956Y | Brugada syndrome |
| *CACNB2* | 600003 | c.428C>T | p.S143F | Brugada syndrome |
| *CALR3* | 611414 | c.245A>G | p.K82R | Cardiomyopathy, hypertropic |
| *CASQ2* | 114251 | c.919G>C | p.D307H | Ventricular tachycardia, polymorphic |
| *CAV3* | 601253 | c.233C>T | p.T78M | Long QT syndrome |
| *CBS* | 613381 | c.1397C>T | p.S466L | Homocystinuria |
| *COL3A1* | 120180 | c.3230G>T | p.G1077V | Ehlers-Danlos syndrome IV |
| *COL5A1* | 120215 | c.1588G>A | p.G530S | Ehlers-Danlos syndrome |
| *COL5A2* | 120190 | c.1933G>A | p.G645R | Ehlers-Danlos syndrome |
| *CRYAB* | 123590 | c.470G>A | p.R157H | Cardiomyopathy, dilated |
| *CSRP3* | 600824 | c.10T>C | p.W4R | Cardiomyopathy, dilated |
| *CTF1* | 600435 | c.274G>A | p.A92T | Cardiomyopathy, dilated |
| *DES* | 125660 | c.1358C>T | p.T453I | Cardiomyopathy, restrictive with atrioventricular block |
| *DMD* | 300377 | c.54G>T | p.K18N | Cardiomyopathy, dilated |
| *DMD* | 300377 | c.5371C>T | p.Q1791* | Muscular dystrophy, Duchenne |
| *DSC2* | 125645 | c.607C>T | p.R203C | Arrhythmogenic right ventricular cardiomyopathy |
| *DSG2* | 125671 | c.2434G>T | p.G812C | Cardiomyopathy, arrhythmogenic right ventricular |
| *DSP* | 125647 | c.2422C>T | p.R808C | Cardiomyopathy, arrhythmogenic right ventricular |
| *DTNA* | 601239 | c.362C>T | p.P121L | Left ventricular noncompaction with CHD |
| *ELN* | 130160 | c.2T>C | p.M1T | Cardiomyopathy & pulmonary emphysema |
| *EMD* | 300384 | c.454C>T | p.R152C | Cardiomyopathy, dilated |
| *FBN1* | 134797 | c.184C>T | p.R62C | Marfan syndrome |
| *FBN2* | 612570 | c.976C>T | p.P326S | Contractural arachnodactyly |
| *FKTN* | 607440 | c.1073A>C | p.Q358P | Cardiomyopathy, dilated |
| *GATAD1* | 614518 | c.304T>C | p.S102P | Cardiomyopathy, dilated |
| *GLA* | 300644 | c.1066C>T | p.R356W | Fabry disease |
| *GPD1L* | 611778 | c.839C>T | p.A280V | Brugada syndrome |
| *HRAS* | 190020 | c.35G>T | p.G12V | Costello syndrome |
| *JPH2* | 605267 | c.421T>C | p.Y141H | Cardiomyopathy, hypertrophic |
| *JUP* | 173325 | c.56C>T | p.T19I | Arrhythmogenic right ventricular dysplasia/cardiomyopathy |
| *KCNE1* | 176261 | c.221C>T | p.S74L | Long QT syndrome |
| *KCNE2* | 603796 | c.161T>C | p.M54T | Cardiac arrhythmia |
| *KCNE3* | 604433 | c.296G>A | p.R99H | Brugada syndrome |
| *KCNH2* | 152427 | c.157G>C | p.G53R | Long QT syndrome |
| *KCNJ2* | 600681 | c.407C>T | p.S136F | Andersen syndrome |
| *KCNJ5* | 600734 | c.1159G>C | p.G387R | Long QT syndrome |
| *KCNJ8* | 600935 | c.1265C>T | p.S422L | Ventricular fibrillation |
| *KCNQ1* | 607542 | c.502G>A | p.G168R | Long QT syndrome |
| *KRAS* | 190070 | c.101C>G | p.P34R | Cardio-facio-cutaneous syndrome |
| *LAMA4* | 600133 | c.3217C>T | p.R1073* | Cardiomyopathy, dilated |
| *LAMP2* | 309060 | c.877C>T | p.R293* | Danon disease |
| *LDB3* | 605906 | c.349G>A | p.D117N | Cardiomyopathy, dilated |
| *LMNA* | 150330 | c.388T>C | p.Y130H | Cardio-facio-cutaneous syndrome |
| *MAP2K1* | 176872 | c.371C>A | p.P124Q | Cardio-facio-cutaneous syndrome |
| *MYBPC3* | 600958 | c.1468G>A | p.G490R | Cardiomyopathy, hypertrophic |
| *MYH11* | 160745 | c.5273G>A | p.R1758Q | Aortic aneurysm, familial thoracic 4 |
| *MYH6* | 160710 | c.3010G>T | p.A1004S | Cardiomyopathy, dilated |
| *MYH7* | 160760 | c.1208G>A | p.R403Q | Cardiomyopathy, hypertrophic |
| *MYL2* | 160781 | c.173G>A | p.R58Q | Cardiomyopathy, hypertrophic |
| *MYL3* | 160790 | c.170C>G | p.A57G | Cardiomyopathy, hypertrophic |
| *MYLK2* | 606566 | c.260C>T | p.A87V | Cardiomyopathy, hypertrophic |
| *MYOZ2* | 605602 | c.738A>G | p.I246M | Cardiomyopathy, hypertrophic |
| *NEXN* | 613121 | c.1955A>G | p.Y652C | Cardiomyopathy, dilated |
| *NRAS* | 164790 | c.149C>T | p.T50I | Noonan syndrome |
| *PKP2* | 602861 | c.1237C>T | p.R413* | Arrhythmogenic right ventricular dysplasia |
| *PLN* | 172405 | c.25C>T | p.R9C | Cardiomyopathy, dilated |
| *PRKAG2* | 602743 | c.1199C>A | p.T400N | Cardiomyopathy, hypertrophic |
| *PSEN1* | 104311 | c.104G>A | p.R35Q | Alzheimer disease & Dilated cardiomyopathy, 1U |
| *PSEN2* | 600759 | c.389C>T | p.S130L | Alzheimer disease & Dilated Cardiomyopathy, IV |
| *PTPN11* | 176876 | c.922A>G | p.N308D | Noonan syndrome |
| *RAF1* | 164760 | c.752T>C | p.L251P | Perinatal problems & hypertrophic cardiomyopathy |
| *RBM20* | 613171 | c.1901G>A | p.R634Q | Cardiomyopathy, dilated |
| *RYR2* | 180902 | c.527G>A | p.R176Q | Arrhythmogenic right ventricular dysplasia type 2 |
| *SCN1B* | 600235 | c.259G>C | p.E87Q | Cardiac conduction disease |
| *SCN3B* | 608214 | c.29T>C | p.L10P | Brugada syndrome |
| *SCN4B* | 608256 | c.485T>G | p.V162G | Atrial fibrillation |
| *SCN5A* | 600163 | c.481G>A | p.E161K | Brugada syndrome |
| *SDHA* | 600857 | c.1664G>A | p.G555E | Complex II deficiency & Dilated cardiomyopathy, 1GG |
| *SGCD* | 601411 | c.451T>G | p.S151A | Cardiomyopathy, dilated |
| *SHOC2* | 602775 | c.4A>G | p.S2G | Noonan-like syndrome with loose anagen hair |
| *SLC25A4* | 103220 | c.368C>A | p.A123D | Mitochondrial myopathy & hypertrophic cardiomyopathy |
| *SLC2A10* | 606145 | c.243C>G | p.S81R | Arterial tortuosity syndrome |
| *SMAD3* | 603109 | c.715G>A | p.E239K | Thoracic aortic aneurysms and dissections |
| *SNTA1* | 601017 | c.770C>G | p.A257G | Long QT syndrome |
| *SOS1* | 182530 | c.1870G>T | p.V624F | Cardiomyopathy, hypertrophic |
| *TAZ* | 300394 | c.589G>A | p.G197R | Barth syndrome |
| *TCAP* | 604488 | c.316C>T | p.R106C | Cardiomyopathy, hypertrophic |
| *TGFB3* | 190230 | c.1150G>A | p.E384K | Cardiomyopathy, arrhythmogenic right ventricular |
| *TGFBR1* | 190181 | c.1058G>T | p.G353V | Loeys-Dietz aortic aneurysm syndrome |
| *TGFBR2* | 190182 | c.923T>C | p.L308P | Marfan syndrome II |
| *TMEM43* | 612048 | c.1073C>T | p.S358L | Arrhythmogenic right ventricular cardiomyopathy |
| *TMPO* | 188380 | c.2068C>T | p.R690C | Cardiomyopathy, dilated |
| *TNNC1* | 191040 | c.476G>A | p.G159D | Cardiomyopathy, dilated |
| *TNNI3* | 191044 | c.433C>T | p.R145W | Cardiomyopathy, restrictive |
| *TNNT2* | 191045 | c.421C>T | p.R141W | Cardiomyopathy, dilated |
| *TPM1* | 191010 | c.742A>G | p.K248E | Cardiomyopathy, non-compaction, left ventricular |
| *TTN* | 188840 | c.40579C>T | p.R13527* | Cardiomyopathy, dilated |
| *TTN* | 188840 | c.55735G>A | p.A18579T | Arrhythmogenic right ventricular cardiomyopathy |
| *TTR* | 176300 | c.250T>A | p.F84I | Cardiomyopathy & late-onset polyneuropathy of lower limbs |
| *VCL* | 193065 | c.2923C>T | p.R975W | Cardiomyopathy, dilated |

^*^Online Mendelian Inheritance in Man, ^†^ Human Genome Variation Society, ^‡^Associated phenotype listed in Human Gene Mutation Database
